# Public Interest in Refractive Diseases and Treatments During the COVID-19 Pandemic: A Google Trends Analysis

**DOI:** 10.7759/cureus.17207

**Published:** 2021-08-15

**Authors:** Rishabh Gupta, Haig Pakhchanian, Rahul Raiker, Masumi Asahi, Neha Raparla, David Belyea

**Affiliations:** 1 Ophthalmology, University of Missouri Kansas City School of Medicine, Kansas City, USA; 2 Ophthalmology, George Washington University School of Medicine and Health Sciences, Washington DC, USA; 3 Ophthalmology, West Virgina University School of Medicine, Morgantown, USA

**Keywords:** coronavirus, google trends, lasik, refractive surgery, public interest, internet search, refractive disease, cataract, covid-19

## Abstract

Purpose

To assess national internet search trends/public interest in refractive diseases and treatments during the first year of the COVID-19 pandemic.

Methods

A Google Trends search for refractive terms was performed during the first year of the COVID-19 pandemic. Refractive terms were divided into disease and treatment terms. Relative search volume (RSV) indices were assessed in the United States from the initial lockdown period (March 1 - June 28), summer reopening period (July 5 - November 1), and winter case surge/vaccine rollout period (November 8 - February 28). A t-test of two independent samples assuming unequal variances was utilized in comparing the pandemic year to pooled data of overlapping weeks between 2016-2019.

Results

The majority of disease and treatment terms showed a significant decrease in RSV during the initial lockdown period (p<0.05). There was a significant increase in RSV for cataract, astigmatism, cataract surgery, and photorefractive keratotomy (PRK) (p<0.05), accompanied by a significant decrease in RSV for contact lens during the summer reopening period. There was a significant increase in RSV for cataract, astigmatism, glasses, and PRK, accompanied by a significant decrease in RSV for hyperopia, keratoconus, contact lens, and LASIK during the winter case surge/vaccine rollout period.

Conclusion

There was a significant decrease in the public interest in refractive diseases and treatments during the lockdown period, accompanied by an increase in interest later in the year. Decreased public interest can lead to delays in care, poorer health literacy, and potentially worse outcomes. Strategies to enhance public interest and care during the pandemic may prove to be beneficial.

## Introduction

The Coronavirus Disease 2019 (COVID-19) virus outbreak was declared a global pandemic by the World Health Organization (WHO) on March 11th, 2020 [1]. This led to a recommendation from national organizations, such as the Center for Medicare & Medicaid Services (CMS), to delay all elective surgeries and procedures [2]. With citizens’ focus shifting toward preventative measures against the virus and adequate treatment of those afflicted, public interest in different facets of healthcare has changed dramatically. At the date of writing, while the virus continues to spread and vaccine rollout continues, the United States (US) has seen a modest decrease in new case rates over time despite the persistence of the disease [3].

For ophthalmologists, the effects of the virus are drastic, as close contact up to 6 inches face to face is necessary for day-to-day clinical interactions, procedures, and surgeries. For refractive specialists, this effect is pronounced further. As the majority of refractive interventions - such as cataract surgery, glasses/contact visits, LASIK, and more - are largely considered elective interventions, many patients were unable to seek refractive care during the spring of 2020 [4].

Throughout the pandemic period, refractive specialists have adapted safety measures to continue seeing patients in clinical settings. During this time, it has become important to assess the change in public interest in refractive diseases and interventions. This information is valuable to reveal how patients' perceptions of refractive ophthalmology are subject to change from infectious disease and whether adaptations to the pandemic have changed public attitudes toward the field.

Google Trends (GT) (Google LLC, Mountain View, California) is a tool that has been utilized in clinical research to investigate public interest and attitudes toward an item of interest. By analyzing Google search results within a country or worldwide, GT data has been shown to represent not only public interest, attitudes, and behaviors but also correlate with epidemiologic outbreaks in some cases [5-8]. We herein utilize the GT database to analyze national internet search trends in refractive diseases and treatments during different time periods of the COVID-19 pandemic.

## Materials and methods

We assessed Google search volume for terms relating to refractive care individually and separated each term into two categories: disease and treatment terms. Google search volume is normalized on a scale from 0 to 100 and represented as relative search volume (RSV). An RSV value of 100 represents the highest search volume over a selected time, while a value of 0 represents the least amount of search volume. Google Trends provides an average weekly RSV value. Search terms were assessed for RSV using filters for region, date, and category. The following search strategy was utilized to assess RSV using the parameters: "United States," “January 1, 2016 to February 28th, 2021," and "All Categories." This ensured that RSV was normalized from 0 to 100 for all searches over all analyzed time periods. Terms with common multiple expressions conveying the same meaning were combined with “+” to better capture search interest. The data were categorized into three time periods during the pandemic were as follows: the initial lockdown period (March 1 - June 28), the summer reopening period (July 5 - November 1), and the winter case surge/vaccine rollout period (November 8 - February 28). These were selected due to observed trends in caseload and government responses to changes in the pandemic.

Search term RSV during each of the three periods was compared to pooled data of overlapping weeks between 2016 and 2019. A t-test of two independent samples assuming unequal variances was utilized to compare the two groups. Comparisons with a two-sided p-value<0.05 were considered statistically significant. The average weekly RSV for terms relating to refractive eye care in 2020-2021 was compared to 2016-2019 by graphical analysis. Computations were conducted on Microsoft Excel 2019 (Microsoft Corporation, Redmond, Washington).

## Results

When querying the GT database for mean RSV, we separated disease terms from treatment terms to better compare public interest between disease and treatment conditions. Data was collected from March 1 to June 30, July 1 to October 31, and November 1 to February 28. These time periods were referred to as the lockdown period, summer reopening period, and winter surge/vaccine rollout period, respectively. 

**Figure 1:**
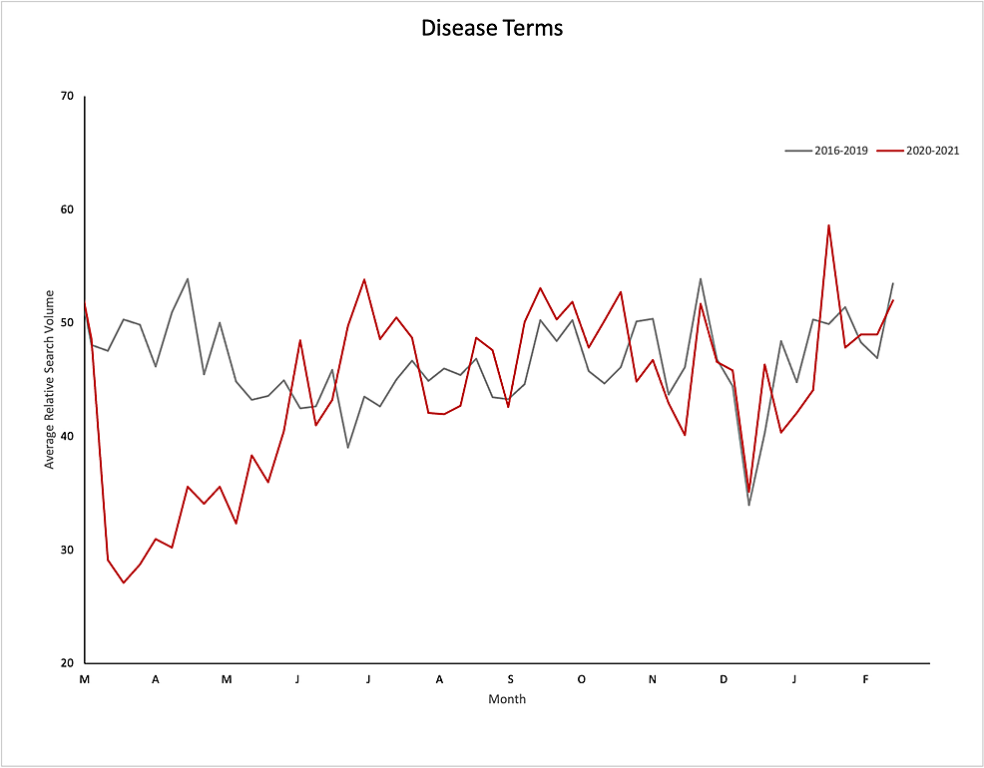
Graphical representation of average relative search volume over time from 2016-2019 (gray line) and 2020-2021 (red line) for selected refractive disease terms in the United States

**Figure 2:**
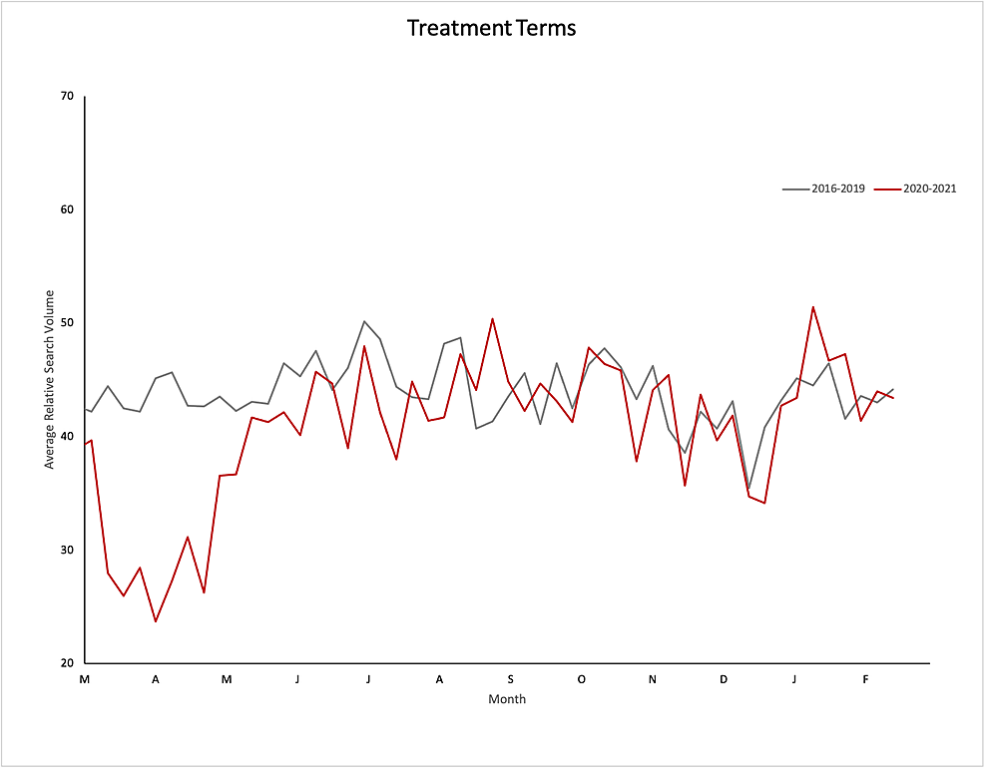
Graphical representation of average relative search volume over time from 2016-2019 (gray line) and 2020-2021 (red line) for selected refractive treatment terms in the United States

Comparing 2016-2019 aggregate mean RSV values with 2020 mean RSV values within the US during each period, disease and treatment terms in order of descending percent change are shown in Table 1. Graphical representations of search interest trends in refractive disease and treatment terms from 2016-2019 and 2020-2021 are shown in Figure 1 and Figure 2. 

**Table 1:** Mean relative search volume for refractive search terms during the COVID-19 pandemic compared to mean relative search volume for refractive search terms throughout similar dates in the years 2016-2019

	March 1 - June 28						July 5 - November 1						November 8 - February 28					
Search term	RSV, 2020, mean (SD)	RSV, 2016-2019, mean (SD)	Percent change	Difference in means	95% CI	P-value	RSV, 2020, mean (SD)	RSV, 2016-2019, mean (SD)	Percent change	Difference in means	95% CI	P-value	RSV, 2020, mean (SD)	RSV, 2016-2019, mean (SD)	Percent change	Difference in means	95% CI	P-value
Disease terms																		
Far Sightedness + Far Sighted	39.8 (19.9)	48.3 (17.4)	-17.70%	-8.5	[-18.6, 1.5]	0.109	43.3 (17.7)	46.8 (17.5)	-7.40%	-3.5	[-12.6, 5.6]	0.462	40.9 (11.9)	47.3 (17.3)	-13.40%	-6.3	[-13.5, 0.9]	0.095
Near Sightedness + Near Sighted	37.8 (10.3)	50 (13.6)	-24.40%	-12.2	[-17.9, -6.5]	<0.001	54 (15.3)	50 (14.9)	8.00%	4.0	[-3.9, 11.9]	0.33	47.4 (18.4)	51.9 (16)	-8.60%	-4.5	[-14.3, 5.4]	0.384
Astigmatism	22.2 (5.4)	27.1 (9.8)	-18.10%	-4.9	[-8.3, -1.5]	0.006	39.1 (15.7)	26.1 (4.2)	49.80%	13.0	[5.7, 20.3]	0.003	32.7 (3.5)	28 (8.2)	16.90%	4.7	[2.1, 7.4]	0.001
Blurry Vision + Blurred Vision	64.1 (6.1)	68.5 (10.9)	-6.40%	-4.4	[-8.1, -0.6]	0.028	65.6 (4.9)	65.7 (8.2)	-0.30%	-0.2	[-3.1, 2.8]	0.905	69.5 (4.6)	68.6 (9.8)	1.30%	0.9	[-2.4, 4.2]	0.586
Cataract	57.3 (20.7)	73.9 (8.7)	-22.50%	-16.6	[-26.4, -6.9]	0.004	87.1 (6.7)	76.2 (10.8)	14.30%	10.9	[6.9, 14.9]	<0.001	81.4 (10.6)	73.1 (12)	11.40%	8.3	[2.3, 14.3]	0.011
Hyperopia	32.7 (9.4)	39.9 (14.9)	-18.00%	-7.2	[-12.8, -1.6]	0.015	30.7 (11.2)	34.6 (13.7)	-11.20%	-3.9	[-10, 2.2]	0.22	35.9 (16.4)	46.1 (18.2)	-22.00%	-10.2	[-19.4, -1]	0.04
Keratoconus	21.4 (8.9)	34.8 (9.9)	-38.50%	-13.4	[-18.1, -8.7]	<0.001	32.4 (5.9)	33.9 (11.5)	-4.20%	-1.4	[-5.2, 2.4]	0.466	28.6 (9.6)	34.9 (9.3)	-18.10%	-6.3	[-11.6, -1.1]	0.027
Myopia	27.1 (5.1)	33 (5.2)	-18.10%	-6.0	[-8.6, -3.3]	<0.001	33.9 (3.7)	34.4 (9.9)	-1.30%	-0.4	[-3.3, 2.4]	0.768	32.9 (6.9)	35 (8.5)	-6.20%	-2.2	[-6.1, 1.8]	0.297
Treatment Terms																		
Contact Lens + Contact Lenses	43.8 (5.1)	53.9 (4.3)	-18.70%	-10.1	[-12.7, -7.5]	<0.001	54.3 (8.7)	62.6 (9.7)	-13.20%	-8.3	[-12.9, -3.7]	0.001	45.8 (3.3)	50.9 (4.5)	-10.10%	-5.1	[-7.1, -3.2]	<0.001
Cataract Surgery + Cataract Removal + Cataract Extraction	49.4 (19.9)	66.4 (12)	-25.60%	-17.0	[-26.6, -7.4]	0.002	78.2 (8)	68.7 (13)	13.80%	9.5	[4.8, 14.3]	<0.001	74.8 (10.2)	69.5 (12.9)	7.60%	5.3	[-0.6, 11.2]	0.091
Glasses + Spectacles + Eyeglasses	15.1 (1.9)	14 (1)	7.20%	1.0	[0.1, 1.9]	0.041	17 (1.1)	17.1 (13.9)	-0.40%	-0.1	[-3.3, 3.2]	0.967	16.9 (0.8)	15.8 (1)	7.40%	1.2	[0.7, 1.6]	<0.001
Intraocular Lens	28 (12.7)	39.6 (15.1)	-29.20%	-11.6	[-18.4, -4.7]	0.002	31.6 (15.8)	37.8 (21.1)	-16.40%	-6.2	[-15, 2.6]	0.174	32.3 (14)	38.3 (16)	-15.70%	-6.0	[-13.9, 1.9]	0.147
LASEK + Laser Assisted Sub Epithelial Keratectomy	37.3 (17.2)	43.6 (16.3)	-14.50%	-6.3	[-15.1, 2.5]	0.173	32.6 (15.1)	41 (19.5)	-20.50%	-8.4	[-16.7, -0.1]	0.055	37.8 (14)	37.4 (16.5)	1.00%	0.4	[-7.6, 8.4]	0.924
LASIK + Laser in Situ Keratomileusis	37.4 (9.6)	53.1 (5.2)	-29.50%	-15.7	[-20.3, -11.1]	<0.001	49.1 (5)	50.7 (5)	-3.30%	-1.7	[-4.3, 0.9]	0.224	50.3 (5.3)	54.6 (9)	-7.80%	-4.3	[-7.7, -0.8]	0.019
PRK + Photorefractive Keratectomy	37.4 (10.7)	37.8 (6.1)	-0.80%	-0.3	[-5.5, 4.8]	0.904	45.3 (6.2)	38.7 (11.2)	17.10%	6.6	[2.8, 10.5]	0.002	39.6 (4.6)	34.2 (6.4)	15.80%	5.4	[2.7, 8.2]	0.001

Disease terms

Disease terms relating to keratoconus, near-sightedness, cataracts, astigmatism, myopia, hyperopia, and blurry vision all saw statistically significant (p<0.05) decreases in RSV when compared to 2016-2019 data during the lockdown period (in order of descending percent change). During the summer reopening period, the terms cataract and astigmatism saw a relative increased RSV, suggesting a substantial rebound in the public interest at this time. During the winter surge/vaccine rollout period, terms relating to hyperopia and keratoconus showed a relative decrease in RSV, while terms relating to cataract and astigmatism showed a relative increase in RSV. This suggests continued decreased public interest in hyperopia and keratoconus and a continued rebound in the public interest for the terms relating to cataract and astigmatism during the winter surge/vaccine rollout period.

Treatment terms

Treatment terms relating to LASIK, intraocular lens, cataract surgery, contact lens, and glasses saw statistically significant decreases in RSV when compared to 2016-2019 data during the lockdown period (in order of descending percent change). During the summer reopening period, the term contact lens showed a relative decrease in RSV, suggesting a continued decrease in the public interest for this term, while the terms cataract surgery and photorefractive keratotomy (PRK) showed a relative increase in RSV, suggesting a rebound in the public interest for these terms. During the winter surge/vaccine rollout period, terms relating to contact lenses and LASIK saw continued relative decreases in RSV compared to prior years, while terms relating to glasses and PRK saw a relative increase in RSV. This suggests a continued decrease in public interest in LASIK and contact lenses and an increase in interest relating to glasses and PRK during the winter surge/vaccine rollout period.

## Discussion

This study found significant trends in internet searches of refractive diseases and treatments from the periods of March 1 to June 28, July 5 to November 1, and November 8 to February 28. These dates correlate with the initial lockdown period, the summer reopening period, and the winter case surge/vaccine rollout period respectively. Our analysis revealed a significant decrease in public interest among select terms during the initial lockdown period, as well as rebounds in the public interest of select terms during the summer reopening and winter surge/vaccine rollout periods.

We hypothesize the likely cause for the relative search volume (RSV) decrease in refractive search terms during the initial lockdown period is the peak of the COVID-19 pandemic, resulting in limited access to elective ophthalmic care and a shift of public attitudes toward the respiratory illness [4]. An example of refractive care disruption is illustrated by Aggarwal et al.’s analysis of the impact on cataract surgery during the COVID-19 pandemic. They suggest that the cataract extraction case backlog may be as high as 1.1-1.6 million through May 2022 [9]. The updated forecast of case backlog is likely much higher at the time of writing, as this recent analysis did not take into account multiple waves of COVID-19 case incidence increases. Hom et al.’s recent study analyzed GT data to understand how patients utilize search engines for information relating to low vision [10]. In terms relating to refraction, such as myopia and cataract, disease education and treatment education were the leading classifications of uses for these internet searches. We infer that barriers in access to refractive care would result in a decrease of refractive internet searches due to a lack of patients seeking disease and treatment education. As the CMS released guidelines on May 6, 2020, allowing for the gradual resumption of elective procedures, it is possible that this resumption of ophthalmic care supported a relative increase in internet searches of some diseases and treatments during the summer reopening and winter surge/vaccine rollout periods.

Our study found that terms relating to cataracts, cataract surgery, and intraocular lenses saw a statistically significant decrease during the lockdown period of 2020. As cataract surgery has been known to be the most commonly performed surgery in the developed world, the stark relative decrease in internet searches relating to cataract surgery during the pandemic comes as a little surprise [11]. While a recent study showed that 64.8% of their 207 patient sample expressed a decreased quality of life due to inability to receive cataract extraction during the pandemic, as much as 47% of the surveyed population described some concern in contracting COVID-19 at the hospital during the early summer of 2020 [12]. Along with barriers to care, this fear of contracting COVID-19 during the initial phase of the pandemic likely contributed to decreased internet queries of cataracts and cataract surgery. Interestingly, our data also suggest that queries for cataract and cataract surgery saw a significant increase during the summer reopening period and winter surge/vaccine rollout period. As procedures resumed during the summer of 2020, proposed solutions to continue safe cataract surgery included measures such as rationing cases based on visual acuity and only seeing eyes, distributing adequate personal protective equipment (PPE) among patients and providers, and incorporating telehealth to reduce postoperative visits for routine cases [13]. While modified cataract surgeries resumed and the COVID-19 caseload decreased during the summer reopening period, increased access to eye care, and reduced patients' concerns may have contributed to the increase in the public interest in cataracts and cataract surgery.

Treatment terms relating to LASIK were found to have consistent decreases in internet search interest during the initial lockdown period in our study. LASIK has historically been subject to public scrutiny due to various press releases and its lack of insurance reimbursement in most cases. A 2013 GT study by Stein et al. reported that the internet query rate for terms relating to LASIK decreased as much as 40% nationally from 2007-2011, and approximately 24% and 22% in India and the UK during this period, respectively [14]. Notably, our study suggests that public interest in LASIK decreased significantly during the initial lockdown and the winter surge/vaccine rollout period, with no recovery in interest throughout the pandemic. Although studies have suggested that the transmission risk of the virus is low given that proper precautions are taken to combat infection risk during the procedure, it is unclear why the public interest in LASIK continued to decrease through the winter period [15, 16]. Though it is likely the elective nature of LASIK contributed to the internet search trends seen in our data, there remains a possibility that public scrutiny and media coverage continue to affect the public interest in the procedure. 

Our findings mirror previous analyses of internet search trends pertaining to symptoms, diseases, and treatments of other medical subspecialties during the onset of the COVID-19 pandemic. Bhambhvani et al. showed a statistically significant decrease in internet search terms relating to elective urological procedures in the pandemic period compared to the pre-pandemic period (March 2020-May 2020, March 2015-May 2020, respectively) [17]. Guzman et al. showed a statistically significant decrease in internet search terms of general dermatologic conditions, dermatologic precancerous and malignant conditions, and dermatologic cosmetic procedures during the pandemic period when compared to pre-pandemic data (March 15, 2020 - March 29, 2020, April 29, 2019 - March 8. 2020, respectively) [18].

GT analysis has also played a role in suggesting ocular manifestation of the coronavirus during the pandemic. Deiner et al. reported an increased search interest in terms representing conjunctivitis and other ocular surface conditions during the spring of 2020, supporting previous literature describing ocular symptoms in patients diagnosed with COVID-19 [19, 20]. Mirza et al. reported a correlation between keywords pertaining to ocular symptoms of COVID-19 and the number of new COVID-19 cases throughout the pandemic [21]. These results together support prior articles suggesting ocular signs and transmission of the coronavirus, likely due inability of the immune system to defend against pathogens in the tear film [22, 23]. 

It is important to consider solutions to implement safe ophthalmic care in the COVID-19 era while also retaining patients’ interest in their ocular health. A recent GT study by Ali et al. describes a positive fair correlation between global interest in telehealth and the number of new COVID-19 cases diagnosed over time [24]. As ophthalmology clinics remain low in patient volume during the pandemic, virtual ophthalmology visits are taking place. Saleem et al. describe methods to monitor patient ophthalmic health, utilizing tools such as mobile apps or printed charts for visual acuity and Amsler grids, and utilization of virtual video calls for preliminary examinations [25]. More accurate methods are also discussed, such as out-of-clinic access to remote slit-lamps, nonmydriatic fundus cameras, and optical coherence tomography machines. Others have also described hybrid telehealth clinics and in-clinic modifications to safely care for patients during the COVID-19 pandemic [26, 27]. Through accommodations like these, the encouragement of telehealth visits and ophthalmic health monitoring may increase patients’ interest in their ocular health. As previous studies show a link between patients’ involvement in their healthcare, compliance, and health outcomes, an increase in telehealth and access to ophthalmic care may likely translate to improved patient care and ocular health outcomes [28, 29]. 

There are several limitations to our study. Firstly, we analyzed all search results collected within the US together, though geographic stratification may alter the significance of our data. Second, we analyzed data utilizing searches in the English language in the US, though many searches may be in a different language. This results in a subset of data pertaining to mostly English-speaking citizens. Lastly, we only utilized the google search engine, though other search engines exist that may provide public users similar information. However, it should be noted that Google retains approximately 92% of the worldwide market share for internet searches [30]. 

## Conclusions

To the best of our knowledge, this is the first study analyzing national internet searches for refractive diseases and treatments during the COVID-19 pandemic and the first to analyze internet search trends across different milestones throughout the pandemic. As previous literature has demonstrated the efficacy of Google Trends studies in gauging public interest, we use this data to compare public interest in refractive ophthalmology from the pandemic to pre-pandemic times. We report a significant decrease in select refractive internet searches during the initial lockdown period (March 1, 2020 - June 28, 2020), as well as an increase in select refractive internet searches through the summer reopening (July 5, 2020 - November 1, 2020) and winter case surge/vaccine rollout (November 8, 2020 - February 28, 2021) periods. This article not only allows us to reflect on past patterns of public interest in refractive diseases and treatments but may also help predict future patterns in the public interest for this topic-particularly as COVID-19 cases rise at the time of writing.
